# Can the return of rural labor improve the physical health of left-behind parents—evidence from rural China

**DOI:** 10.3389/fpubh.2024.1393419

**Published:** 2024-07-10

**Authors:** Huinan Ge, Shu Bian, Zhihan Wang, Zilong Wang

**Affiliations:** ^1^School of Public Administration, Liaoning University, Shenyang, China; ^2^Liaoning Vocational and Technical College of Economics, Shenyang, China

**Keywords:** rural labor return, parental health, multiple linear regression, PSM, IV-2SLS

## Abstract

Objectively In objective terms, the return of rural labor force shortens the spatial distance with parents, leading to changes in caregiving support, emotional support, and financial support for parents, thereby affecting the health status of parents. This article, using data from the Chinese Family Panel Studies, analyzes the characteristics of the health status of parents with and without returning migrant children. By employing multiple linear regression models, PSM models, and IV-2SLS methods to address endogeneity bias, the study preliminarily explores the impact of rural labor force return on parental health. The results show that: (1) among the 5,760 older adult individuals, 1866 of them have returning migrant chil-dren, while the remaining 3,894 do not have returning migrant children. (2) Parents’ health status generally follows a normal distribution, with a small proportion of parents having very poor or very good health. The proportions of parents with relatively poor, fair, and relatively good health status range between 20 and 40%. Among parents with returning chil-dren, 40.12% have relatively poor health status, 45.01% have fair health status, and a small proportion have very poor or very good health status. In contrast, among parents without returning children, the proportions of parents with relatively poor, fair, and rela-tively good health status are 21.69, 33.21, and 38.45%, respectively. When parents tran-sition from not having returning children to having returning children, their health status decreases by 0.541 levels, indicating a negative impact of rural labor force return on par-ents’ health. Based on the analysis results, this article provides policy recommendations from three aspects: how to increase the income of returning labor force, improve the rural pension system, and enhance the concept of children supporting their parents.

## 1 Introduction

The overall change in the mobility of rural labor can be divided into three stages. From the late 1950s to the late 1970s, due to strict restrictions imposed by government policies, there was almost no mobility of labor between urban and rural areas in China. In 1978, rural economic reforms began, and in 1984, the Central Document Number One explicitly granted farmers a certain degree of autonomy in choosing employment. Since the 1990s, there has been a large influx of rural labor force into cities, leading to the formation of the “migrant worker phenomenon.” By the mid to late 1990s, with the reform of state-owned enterprises and the adjustment of economic structure, the number of employment opportunities in cities decreased, resulting in increased employment pressure. As a result, rural labor force was forced to return to their hometowns, leading to an increase in the scale of return migration (1, 2). In 2008, the outbreak of the financial crisis led to an expansion in the scale of rural labor force returning to their hometowns. In recent years, with the shift of industries from the eastern coastal areas to the central and western regions of China, the rapid development of the economy in these regions has made the phenomenon of migrant workers returning or returning to their hometowns even more evident.

According to the “2011–2016 Migrant Worker Monitoring and Survey Report” released by the National Bureau of Statistics (1), it can be seen that from 2011 to 2016, the growth rate of the total number of migrant workers slowed down, gradually decreasing from 3.4% in 2011 to 0.3% in 2016. Based on the reading of the “2015–2018 Migrant Worker Monitoring and Survey Report” (2), it is evident that the growth rate of migrant workers was consistently lower than that of local workers during this period.

By 2017, the aging process of the rural population in China had reached 15%. This means that in rural areas, the population aged 65 and above accounted for 15% of the total rural population, surpassing the threshold of 7% for an aging society. The issue of older adult care in rural areas deserves attention. The traditional older adult care model in rural areas mainly relies on family caregiving, specifically referring to adult children living with their older adult parents and providing them with financial support, care, and emotional comfort (3). However, with a large number of young and middle-aged rural labor force migrating to cities, there has been a significant increase in left-behind older adult individuals in rural areas. As a result, the model of relying on family caregiving for the older adult in rural areas has become increasingly fragile. After the children migrate for work, there is a significant decrease in the emotional support received by the older adult, leading to a notable decline in their quality of life (4–7). Moreover, the financial support provided by their working children does not significantly improve the parents’ quality of life (8, 9). Additionally, rural older adult individuals often have lower incomes, and the cost of relying on others for care is too high. As a result, the health of rural older adult people is difficult to guarantee. Part of the literature suggests that children working outside the home improve their parents’ health (10–12).

With the slowdown in the growth rate of migrant rural workers and the gradual return of rural labor force, will the health of rural older adult people improve? Studying the impact of rural labor force returning on the health of parents is beneficial for providing reference basis in formulating rural older adult care policies for the country and promoting the improvement of the rural older adult care system.

## 2 Literature review

### 2.1 Study on the impact of children’s migration for work on parental health

A large number of young and capable rural laborers are flowing into cities, leading to the serious issue of “hollowing out” in rural areas. Many older adult people are unable to receive basic care and often have to take on more agricultural labor and provide intergenerational support, such as raising grandchildren. In China, the older adult care system is primarily based on the family, and the support provided to older adults mainly includes financial assistance, daily care, and emotional comfort (13, 14). All three factors, namely financial support, daily care, and emotional comfort, have a crucial impact on the quality of life for older adults. The health condition of the older adult is considered a key determinant of their quality of life. Currently, there is no consensus in the academic community regarding the impact of children’s migration for work on the health of older adults, based on the existing research.

Some studies argue that children’s migration for work can promote the health of older adults. For example, the research conducted by Tang et al. (15) found that migrant children can provide increased financial support to their parents, offsetting the negative impact of reduced caregiving on the health of parents. Lin et al. (16), Thapa et al. (17) conducted a survey research in Thailand and found that left-behind older adult individuals have more opportunities to access healthcare services compared to non-left-behind older adult individuals. The increased financial support provided by children’s migration for work is beneficial for the physical health of older adults. Bridges et al. (18) utilized the data from CHARLS in 2011 and empirically demonstrated through Probit model and two-stage least squares that children’s migration for work improves the health status of older adults in rural areas.

Some studies argue that children’s migration for work has negative impacts on the health of older adults. Firstly, the migration of adult children disrupts traditional filial culture and undermines the older adult’s reliance on family support for their older adult care, which may be detrimental to their health. As Willis et al. (19) suggested in his study on left-behind older adult individuals in Indonesia, migrant children are influenced by Western individualistic values and gradually abandon traditional caregiving concepts. This leads to a decrease, rather than an increase, in the support received by older adults from their children. Jingzhong et al. (20) found that the increased financial support provided by children’s migration for work is relatively low, and there is a situation where parents have to take care of their grandchildren, indirectly increasing the financial burden on parents. He et al. (21),Lam et al. (22) found that in Albania, young people who migrate for work do not necessarily provide financial support to their left-behind partners or older adult parents. Secondly, children’s migration for work leads to a reduction in caregiving support and emotional support to parents, which negatively impacts their health. Bhandari et al. (23), Knodel et al. (24), Zhou et al. (25) utilized an instrumental variable Oprobit model and found that children’s migration for work resulted in a decline in the health status of parents. Geist et al. (26), Mosca et al. (27), Donato et al. (28), Adhikari et al. (29) used an Iv-probit model and confirmed that parents’ health status declines after their children migrate for work. Jingzhong et al. (20), Lahaie et al. (30), Hoang et al. (31) conducted a multiple linear regression analysis and employed PSM analysis for robustness testing. They found that after children migrate for work, there is a reduction in the caregiving and emotional support provided by children to their parents, leading to a decline in the health status of older adults. However, some scholars argue that the advancement of modern communication technology and the internet can significantly alleviate the negative effects on the emotional comfort experienced by older adults as a result of their children migrating for work (32–36).

### 2.2 Research on the return of rural labor force

The issue of rural labor force return has received significant attention from the academic community. Existing research mainly focuses on two aspects: On one hand, some literature analyzes the reasons for the return or non-return of rural labor force from individual factors, family factors, and macroeconomic factors. Jensen et al. (37) argue that the choice of rural labor force return is influenced by factors such as family factors, human capital, and local job opportunities. Zhao et al. (38), Razavi et al. (39), Macaísta Malheiros et al. (40) conducted empirical research on the impact of long-term social security on the decision of rural labor force return and concluded that the lack of long-term social security in urban areas is the main reason for rural labor force to return in the long term. Zhang et al. (41), Hao et al. (42) conducted research on rural labor force return intention, taking the background of rural revitalization into consideration. The study combined individual factors, family factors, and agricultural factors to examine the factors influencing the intention of rural labor force to return.

On the other hand, some literature focuses on the issues of reemployment and entrepreneurship after rural labor force return. Liao et al. (43) explored the impact of reemployment after rural labor force return, as well as gender differences, using data from seven provinces. Van Der Sluis et al. (44) argues that family factors have a more prominent influence on rural labor force returning for entrepreneurship compared to economic gains and status attainment. Van Praag et al. (45) studied the factors affecting the performance of rural labor force returning for entrepreneurship. There is limited literature that specifically investigates the impact of rural labor force return on the health of parents.

### 2.3 Research on the impact of rural labor force return on parental older adult care

The impact mechanism of rural labor force return on parental older adult care is similar to the mechanism of children’s migration for work affecting parental older adult care. It primarily affects the parents’ older adult care situation in terms of economic support, daily care, and emotional comfort. For example, Maher et al. (46), Kobeissi et al. (47) utilized field survey data from three provinces and demonstrated that the economic support, daily care, and emotional comfort provided by returning children to their parents improved the parents’ quality of life to varying degrees. Harrison et al. (48), Constant et al. (49) from the perspective of human capital, conducted an analysis and found that the accumulation of human capital among returning rural migrant workers is beneficial for them to provide better emotional comfort and economic support to their parents.

### 2.4 Comments

Based on the literature analysis mentioned above, two shortcomings can be identified: Firstly, there is limited research on the impact of rural labor force return on rural older adult care in the existing literature. Secondly, with the popularization of the new agricultural insurance policy, the health status of older adult people will be affected to a certain extent. However, in the literature studying the impact of children’s migration for work on the health of older adult people, there is limited inclusion of the amount of rural older adult pension in the research indicator system. Studying the relevant issues is beneficial for the government to formulate rural pension policies based on practical reference, and it promotes the health of middle-aged and older adult people in rural areas. Therefore, based on the actual survey data from the “China Rural Survey” in 2018, this study includes the monthly pension amount in the indicator system measuring the socio-economic characteristics of the older adult, and investigates the impact of migrant workers returning to the rural areas on the health of their parents. The aim is to make a contribution in this area.

## 3 Theoretical analysis and research hypotheses

In traditional agricultural societies, the entire family serves as the production and consumption unit, and social welfare systems are not widespread. Therefore, older adult individuals mainly rely on family care for their old age. According to traditional beliefs, adult children live with their parents, providing emotional and caregiving support, sharing in household chores to ensure the welfare and health of the older adult. When adult children leave home, parents may take on more agricultural activities and help raise grandchildren, negatively impacting their own health (1). Therefore, when the workforce returns home, it may reduce the spatial distance between parents and provide them with more emotional comfort and labor support.

However, some researchers argue that with the advancement of various communication methods, even when adult children work outside the home, they can still interact with their parents through various means of communication, providing emotional comfort. Therefore, when adult children return home, it may not necessarily bring additional benefits to the health of their parents in this aspect. Additionally, according to the new economics of migration theory, the migration of adult children for work can help diversify the economic risks of the family, ultimately having a positive impact on the family. According to this theory, adult children working outside the home can increase the family’s income, access more health information, and provide more economic support and scientifically sound health advice to ensure the physical health of their parents. Parents can use these resources to improve their standard of living and access better medical resources such as regular check-ups and treatment for chronic illnesses.

**Figure 1:**
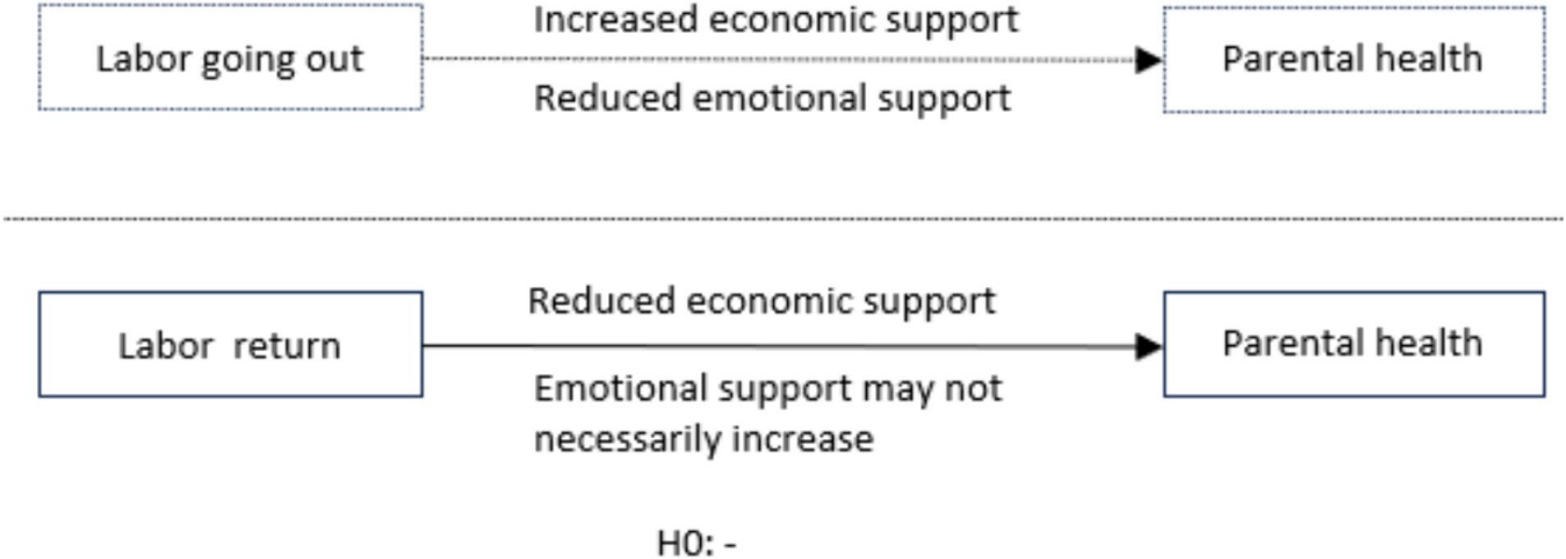
Conceptual model diagram.

When rural labor returns home, it may to some extent weaken the economic support for parents, reduce the opportunity for parents to access resources beneficial to their health, thus negatively affecting the health of parents. Furthermore, when adult children return home, their own income decreases, leading to a lower standard of living. In situations where parents allow it, they may even encroach on the parents’ financial resources. Additionally, with more frequent contact with parents, there is a higher likelihood of friction due to generation gaps and lifestyle differences, which can have a negative impact on the parents’ health, the above relationships can be simplified and seen in Figure 1. In conclusion, this paper proposes the research hypothesis:

Hypothesis H1: The impact of the return of rural labor force on the health status of parents is negative.

## 4 Research design

### 4.1 Data source

The data source of this article is the “China Family Panel Studies” (CFPS) conducted by the China Family Tracking Survey Center at Peking University. This database conducted a nationwide baseline survey starting from 2010, with follow-up surveys conducted every 2 years. The survey covers 25 provinces making it a national survey in China. The project collects data at the individual, family, and community levels, including information on basic family demographics, health status, and population mobility. This article uses the CFPS data from the years 2012, 2014, 2016, and 2018 as the sample data.

### 4.2 Variable settings

#### 4.2.1 Dependent variable

This article is based on the self-rated health status of parents in the CFPS questionnaire. Up to now, numerous studies have demonstrated the effectiveness of using self-rated health as a variable to measure health and predict mortality (50–52). This allows “How would you rate your own health?” to serve as a measure for assessing the health status of older adults. According to the answers, “Very poor,” “Poor,” “General,” “Good,” “Very good” were assigned to“1–5,” the higher the score, indicating better health.

#### 4.2.2 Independent variable

Whether there is a backflow child variable. The current academic consensus on the definition of rural labor force inflow remains ambiguous. Xu et al. (53), Djafar et al. (54), Kousis et al. (55) defines “returning migrant workers” as rural residents who have worked outside their household registration area (county-level city) for more than 6 months since 2007 (including 2007), and have returned to their registered residence at the county, township, or village for more than 1 year without going out again. Yu et al. (56), Jia et al. (57) defines “returning labor force” as rural residents who have worked outside their hometown (including county towns and rural areas) for at least 1 year and have returned to their hometown for at least 1 year. Nelson et al. (58), Parutis et al. (59) define migrant workers as “rural laborers who have been working outside their hometown for more than 1 year and have returned to their hometown for more than 6 months.” According to previous studies and considering the context of this study, in this research, we define returnee workforce as “rural residents who have had continuous experience of working outside their county for more than a year, starting from the year after 2007 (excluding 2007), and have returned to their county for a year or longer.”

In terms of specific data processing, firstly, individuals aged 45 and above were selected from the family member questionnaire to form the middle-aged and older adult group. Information such as the age, education level, and living situation of their children was obtained based on the family member relationship table. Secondly, the residential addresses of the children from two survey years were consolidated, and by comparing the distance from home, it was determined whether any children had returned. When there was a return of labor force, it was assigned a value of 1, and a value of 0 was assigned when there was no return. Referring to the study by Chan et al. (60) the core explanatory variable in this article is defined as “whether there is at least one adult child from the same household who has returned, “assigning a value of “1″ if “yes” and a value of “0″ if “no.”

#### 4.2.3 Control variables

Through reviewing many similar empirical studies, it has been found that the individual characteristics of respondents and household characteristics also affect the health status of the older adult. In order to avoid the impact of omitted variable bias on the estimation results of the model. This study refers to the research of Logan et al. (61), Lassar et al. (62), Miethe et al. (63), and Caliendo et al. (64) also includes some variables that represent individual characteristics and household characteristics as control variables in the model. The individual characteristics include the gender, age, educational background, cohabitation with a suitable spouse, presence of chronic illness, participation in medical insurance, engagement in agricultural or non-agricultural labor, and self-care ability of the household head. Additionally, at the family level, we selected variables such as household size, average education level of children, average age of children, and household income. Furthermore, we controlled for provincial regions and survey years. Table 1 the meanings and assignments of variables.

**Table 1:** Variable description table.

Variable type	Variable name and description
The interpreted variable	Self-rated health (1 = poor, 2 = fair, 3 = average, 4 = good, 5 = excellent)
Explanatory variable	Whether there is at least one returning child (1 = yes, 0 = no)
Individual characteristic variable	Gender (1 = male, 0 = female)
	Age (year)
	Years of education (1 represents illiteracy, 2 represents primary school, 3 represents junior high school, 4 represents high school, and 5 represents university)
	Is it suitable for spouses to live together (1 = yes, 0 = no)
	Whether suffering from chronic diseases (1 = yes, 0 = no)
	Whether to participate in medical insurance(1 = yes, 0 = no)
	Whether participating in agricultural or non-agricultural work(1 = yes, 0 = no)
	Self care ability in daily life (The number of projects that cannot be completed by oneself in daily activities of 7 projects)
Family characteristic variables	Number of children in the family
	Average age of children
	Average education level of children
	*Per capita* household income(Thousand yuan)
Provincial location	Provincial level dummy variables

### 4.3 Quantitative model

#### 4.3.1 Multiple linear regression model

In order to examine the impact of rural labor force return on parental health, we first construct a set of multiple linear regression models. The model is formulated as follows Equation (1):


(1) $$\mathrm{health}=\beta_{0}+\beta_{1}\mathrm{children}+\beta_{1}\mathrm{controls}+\mu$$

In this case, “health” is the dependent variable, representing the self-rated health status of parents. “children” is the independent variable, indicating whether or not there is at least one returning child. “control” represents a series of control variables mentioned earlier.

Whether children return is a self-selecting behavior and not randomly generated. If effective measures are not taken to handle this behavior, it could lead to an incorrect assessment of the impact of children returning on the parents’ health. In this regard, this study adopts the propensity score matching (PSM) method to mitigate the potential endogeneity issues that may exist in the model, with the aim of obtaining unbiased estimation results.

PSM includes three types of average treatment effects during the calculation process, namely the average treatment effect on the treated (ATT), the average treatment effect (ATE), and the average treatment effect on the untreated (ATU). In general, scholars are interested in ATT, which in this study refers to the health changes experienced by individuals in the group with returning children due to their children’s return. PSM typically calculates the average treatment effect through the following steps: First, following the counterfactual analysis framework proposed by (65), the sample is divided into a treatment group (with returning children) and a control group (without returning children). Therefore, the average treatment effect on the treated (ATT) in the group with returning children can be defined as Equation (2):


(2) $$ATT=E\left\{EY_{1i}/D_{i}=1,\;p\left(X_{i}\right)-EY_{0i}|D_{i}=0,\;p\left(X_{i}\right)\right\}$$

In the formula, represents the result of the treatment group, which is the physical health status of the older adult with returning children, and represents the result of the control group, which is the health status of the older adult without returning children; 𝐷_𝑖_ =1 indicates the presence of returning children, and 𝐷_𝑖_ =0 indicates the absence of returning children. 𝑝 (𝑋_𝑖_) is the propensity score, indicating the probability of the older adult having returning children, estimated by the Logit model. There are two main methods for propensity score matching. One method is nearest neighbor matching, where samples from the reference group that are closest to the individuals in the treatment group are matched, and a simple arithmetic average is computed to obtain the matching result. Within this, there are k-nearest neighbor matching, which involves finding the k nearest individuals in terms of propensity scores from different groups, and caliper matching (also known as radius matching), which limits the absolute distance of propensity scores. Another approach is the global matching method, where the matching results for each individual consider all individuals from different groups. Generally, individuals outside the common support region are excluded. Then, different weights are assigned based on the distance between individuals. Individuals with closer distances have higher weights, while those with larger distances have smaller weights. When the distance exceeds a certain range, the weight can be reduced to zero. If a kernel function is used to calculate the weights, it is referred to as kernel matching.

Next, the samples are matched based on the magnitude of their propensity scores, and the average treatment effect on the treated (ATT) is calculated using the matched samples to assess the impact of returning children on parental health. To ensure the robustness of the results, this study initially employs the nearest neighbor method for matching, followed by matching using both the caliper and kernel methods.

## 5 Analysis of empirical results

### 5.1 Descriptive statistical analysis

Using Stata to conduct descriptive statistical analysis on 10,153 samples, the results are shown in the table below. Table 2 presents the descriptive statistics for all variables, mainly reporting the means of each variable. The first column shows the statistics for all samples, the middle two columns display the statistics for families with returning children and families without returning children, and the last column shows the mean differences between families with and without returning children. Overall, it is observed that there are a total of 3,289 families with returning children. The average age of parents is around 57.978 years, with an average education level equivalent to primary school. The majority of parents cohabit with their spouses, have a high participation rate in insurance, and are primarily engaged in labor activities. In terms of self-care ability, most parents can independently complete daily activities. Regarding family characteristics, families typically have 1 to 2 children. The average age of children is around 30 years, and their educational level ranges from high school to college. The average household income is 9,000 yuan *per capita*. By examining the mean differences, it can be observed that compared to families without returning children, parents in families with returning children are younger, their children are also younger, and the family size is larger.

**Table 2:** Descriptive statistics of variables.

Variable name	All samples-mean	Returned children-mean	No returned children-mean	Meandiff
	(1)	(2)	(3)	(2)–(3)
Whether there is at least one returning child	0.324	–	–	–
Gender	0.490	0.478	0.496	−0.018*
Age	57.978	57.230	58.340	−1.110***
Years of education	2.108	2.078	2.123	−0.045*
Is it suitable for spouses to live together	0.941	0.948	0.937	0.011*
Whether suffering from chronic diseases	0.247	0.239	0.251	−0.012
Whether to participate in medical insurance	0.922	0.923	0.922	0.001
Whether participating in agricultural or non-agricultural work	0.779	0.787	0.775	0.012
Self care ability in daily life	0.236	0.257	0.225	0.032*
Number of children in the family	1.761	1.874	1.707	0.167**
Average age of children	30.594	29.806	30.970	−1.164***
Average education level of children	3.464	3.352	3.517	−0.165*
*Per capita* household income	9.202	9.121	9.240	−0.119

Note: *, **, and *** respectively indicate significant at the 10%, 5%, and 1% levels.

**Figure 2:**
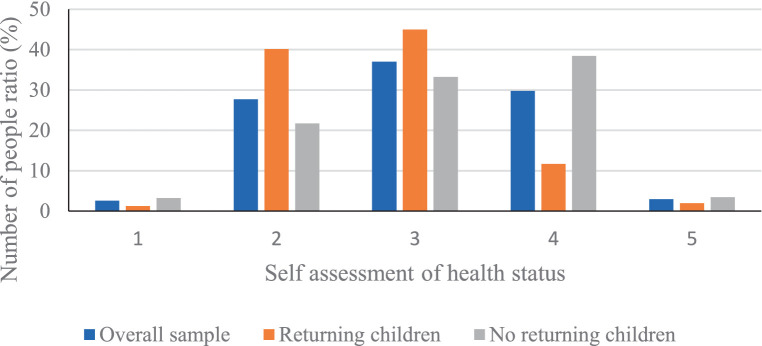
The differences in health status between parents with and without returning migrant child.

In order to better study the impact of children returning on the health status of parents, Figure 2 depicts the differences in parental health status between two groups of samples with and without returning children, as well as the overall distribution of parental health status. From Figure 2, it can be seen that the overall distribution of parental health status is relatively uniform, with a small proportion of parents having very poor or very good health. The proportions of parents with relatively poor, fair, and relatively good health status range between 25 and 40%. The distribution of parental health status for parents with returning children falls between 2 and 4, indicating a range from relatively poor to relatively good health, without instances of very poor or very good health status. For families with returning children, the proportion of parents with relatively poor health status is 40.12%, while those with fair health status make up 45.01%, and the combined proportion of parents with very poor or very good health status is 3.19%. On the other hand, for parents without returning children, the distribution of health status ranges from 1 and 5, with a corresponding number of individuals in each category. The proportions of parents with relatively poor, fair, and relatively good health status range between 20 and 40% in each category. Notably, the proportion of parents with relatively good health status at 38.45% is higher than the 11.67% in families with returning children, while the proportion of parents with relatively poor health status at 21.69% is lower than the 40.12% in families with returning children.

Based on the comprehensive descriptive statistical analysis results mentioned earlier, the return of children may reduce the economic support provided to parents, potentially requiring parents to provide some economic support in return. This situation may also increase possible family friction. Overall, these factors may lead to negative impacts on the health status of parents.

### 5.2 Analysis of the results of the multiple linear regression model

We first constructed a multiple linear regression model to test whether having at least one returning child has an impact on parents’ self-rated health status. The regression was conducted using Stata software, and the results are presented in the table below. From the Table 3, it can be observed that when parents have returning children, their self-rated health status decreases by 0.541, a statistically significant result at the 1% level. Among the control variables, gender, employment status, number of children, average age of children, and household income are all statistically significant at the 1% level, with positive coefficients. This indicates that gender and marital status have a positive impact on the older adult’s self-rated health status, while age, presence of chronic diseases, self-care ability, and self-rated physical health have negative effects.

**Table 3:** Regression results of the multiple linear model.

Variable name	Coef	Robust Std.Err.	*t*
Whether there is at least one returning child	−0.541***	0.048	−11.271
Gender	0.121***	0.031	3.903
Age	−0.014***	0.001	−14.000
Years of education	−0.051	0.045	−1.133
Is it suitable for spouses to live together	0.015	0.041	0.366
Whether suffering from chronic diseases	−0.854***	0.074	−11.541
Whether to participate in medical insurance	0.016	0.024	0.667
Whether participating in agricultural or non-agricultural work	0.201***	0.032	6.281
Self care ability in daily life	−0.145***	0.054	−2.685
Number of children in the family	0.056***	0.013	4.308
Average age of children	0.019*	0.011	1.727
Average education level of children	−0.007	0.011	−0.636
*Per capita* household income	0.054***	0.013	4.154
_cons	7.451***	0.914	8.152
Province	Yes		
Year	Yes		
Obs	5,760		
R^2	0.184		

Note: *, **, and *** respectively indicate significant at the 10%, 5%, and 1% levels.

### 5.3 Analysis of the matching results of propensity score matching (PSM)

After taking the logarithm of the monthly pension amount and the present value of the family’s real estate, PSM analysis was conducted using Stata software. The analysis used the k-nearest neighbor matching method, with k values set to one, two, three, and four for experimentation. Ultimately, *k* = 4 was chosen, representing one-to-four matching. The results are presented in Table 4. From Table 4, it can be observed that ATT represents the average treatment effect for the treated group. The value of ATT is −0. 446, with a t-value of −4.092,this indicates significance at the 1% level, suggesting that the impact of returning migrant children on parents’ health status is negative. In other words, the increase in caregiving support and emotional comfort cannot fully compensate for the reduction in economic support. Considering the results of descriptive statistical analysis, existing theoretical research, the following reasons can be identified:

(1)  Older adult parents have low income, are more susceptible to illness, and have significantly higher expenses. Strengthening economic support plays a crucial role.
(2)  Mutual support and caregiving among parents weaken the role of caregiving support and emotional comfort provided by their children.

**Table 4:** Average treatment effect of children’s return on parents’ health status.

Variable	Sample	Treated	Controls	ATT	S.E.	T-stat
Health	Unmatched	2.784	3.005	−0.221	0.145	−1.524
	ATT	2.675	3.121	−0.446***	0.109	−4.092

Note: *** respectively indicate significant at the 1% levels.

Among the selected sample in this study, a higher proportion of older adult individuals have spouses. The mutual support and comfort provided by the spouses weaken the role of caregiving support and emotional comfort provided by their children.

(3)  The advanced means of communication weaken the impact of increased emotional comfort provided by children after their return.

With the advancement of various communication methods, even when children are working away from home, they can still interact with their parents through various means of communication, providing emotional comfort. Therefore, when children return, they may not necessarily bring additional benefits to their parents’ health status in terms of emotional support, as it can be achieved to some extent through communication regardless of physical proximity.

(4)  The decrease in income for children after their return may encroach upon the financial resources of their parents.

After children return, their own income may decrease, leading to a lower standard of living. In situations where their parents allow it, this decrease in income may potentially encroach upon their parents’ financial resources. Additionally, with more frequent and closer interactions with their parents, issues related to generational gaps and lifestyle differences can arise, leading to conflicts that negatively impact their parents’ health status. In conclusion, the return of children has a negative impact on the health of parents, with hypothesis H0 being supported.

### 5.4 Robustness test

#### 5.4.1 Radius matching

PSM analysis using Stata software was performed with radius matching, defining a radius of 0.01. The results, shown in the graph below, are similar to those of the 4 nearest neighbor matching. The value of ATT is −0. 386, with a *t*-value of −3.299, indicating significance at the 1% level (Table 5).

**Table 5:** Robustness test results of radius matching.

Variable	Sample	Treated	Controls	ATT	S.E.	T-stat
Health	Unmatched	2.784	3.005	−0.221	0.145	−1.524
	ATT	2.625	3.011	−0.386***	0.117	−3.299

Note: *** respectively indicate significant at the 1% levels.

#### 5.4.2 Kernel matching

PSM analysis using the default kernel matching method in Stata software was performed. The results, shown in the graph below, are similar to those of the two previous matching methods. The value of ATT is −0.445, with a *t*-value of −0.099, indicating significance at the 1% level (Table 6).

**Table 6:** Robustness test results of default kernel matching.

Variable	Sample	Treated	Controls	ATT	S.E.	T-stat
Health	Unmatched	2.784	3.005	−0.221	0.145	−1.524
	ATT	2.709	3.154	−0.445***	0.099	−4.495

Note: *** respectively indicate significant at the 1% levels.

In conclusion, the results of the PSM analysis are robust, indicating that the return of children has a negative impact on the health of parents. Therefore, hypothesis H1 is supported (Table 7).

**Table 7:** IV-2SLS result.

	The first stage		The second stage	
Variable name	Coef	Robust Std.Err.	Coef	Robust Std.Err.
IV-Unemployment	0.843***	0.218		
Whether there is at least one returning child			−0.394***	0.012
Gender	0.154	0.214	−0.171***	0.021
Age	0.014**	0.007	0.017***	0.004
Years of education	−0.078	0.945	−0.042	0.039
Is it suitable for spouses to live together	0.015	0.041	0.011	0.009
Whether suffering from chronic diseases	0.054***	0.014	0.541***	0.090
Whether to participate in medical insurance	0.016**	0.008	0.045	0.781
Whether participating in agricultural or non-agricultural work	−0.471***	0.007	−0.341***	0.074
Self care ability in daily life	0.194***	0.049	0.241***	0.041
Number of children in the family	0.056	0.041	−0.043***	0.016
Average age of children	−0.471	0.671	−0.016*	0.009
Average education level of children	0.004**	0.002	−0.009	0.011
*Per capita* household income	0.641***	0.115	−0.078***	0.026
_cons	3.451***	0.784	9.121***	0.845
Province	Yes			
Year	Yes			
Obs	5,760			
*F*-value	25.121			

Note: *, **, and *** respectively indicate significant at the 10%, 5%, and 1% levels.

#### 5.4.3 Endogeneity discussion

Through the theoretical framework discussed earlier, we have found that whether migrant children return home needs to consider the maximization of family utility, which is determined by various factors such as family characteristics and individual traits, including parents’ health status. This indicates a clear bidirectional causal relationship between parents’ health status and the return of migrant children. Specifically, the return of migrant children can alter their older adult support for parents and impact the health status of parents, which is the focus of our study. At the same time, parents’ health status can also influence the decision of migrant children to return. When parents are in poor health, migrant children may choose to continue working outside to cover medical expenses. Alternatively, when parents’ health is deteriorating, migrant children may opt to live closer to provide more daily care or emotional support. Therefore, the endogeneity issue is a crucial consideration when studying the impact of migrant children’s return on parents’ health status. This study will introduce instrumental variables into the ordinary least squares (OLS) regression model to mitigate estimation biases caused by endogeneity.

The instrumental variable chosen in this study is the “urban unemployment rate” at the provincial level. The reason for selecting this variable is that in areas with high urban unemployment rates, there are fewer non-agricultural job opportunities, and returning migrant workers face more severe employment challenges, making them more likely to return. The urban unemployment rate can affect the decision of migrant children to return, but it is essentially unrelated to the health status of parents within individual families, thus meeting the exogeneity requirement and serving as an instrumental variable in this study.

The first stage involves regressing the endogenous explanatory variable of migrant children’s return with the instrumental variable of urban unemployment rate and other exogenous explanatory variables to analyze the relevant characteristics of families with returning migrant children. The results show that the first-stage instrumental variable has an *F*-value of 25.121, significantly exceeding the critical value of 10, indicating the effectiveness of the instrumental variable. Additionally, the coefficient of the urban unemployment rate is significantly positive at the 1% level, suggesting that as hypothesized earlier, a higher urban unemployment rate increases the likelihood of migrant children returning. This could be due to facing greater economic challenges in areas with high urban unemployment rates, leading migrant children to choose to return for their own benefit.

The second stage involves regressing the fitted values of various health assessment indicators on migrant children’s return with all other exogenous explanatory variables. The coefficient for the change in parents’ self-rated health in relation to migrant children’s return is −0.394 and is significant at the 1% level. The regression results indicate that, in terms of subjective health, when migrant children return, parents’ self-rated health significantly decreases, and parents perceive their health to have worsened compared to the past. Overall, the return of migrant children has a negative impact on the health status of parents.

## 6 Main findings and recommendations

### 6.1 Conclusion

This study, using data from the Chinese Family Panel Studies (CFPS), examines the impact of rural labor force return on the health status of parents through descriptive statistics, multiple linear regression models, and PSM models. The findings are as follows:

(1) Among the 5,760 older adult individuals, 1866 of them have returning migrant children, while the remaining 3,894 do not have returning migrant children. (2) Parents’ health status generally follows a normal distribution, with a small proportion of parents having very poor or very good health. The proportions of parents with relatively poor, fair, and relatively good health status range between 20 and 40%. Among parents with returning children, 40.12% have relatively poor health status, 45.01% have fair health status, and a small proportion have very poor or very good health status. In contrast, among parents without returning children, the proportions of parents with relatively poor, fair, and relatively good health status are 21.69, 33.21, and 38.45%, respectively. When parents transition from not having returning children to having returning children, their health status decreases by 0.541 levels, indicating a negative impact of rural labor force return on parents’ health.

### 6.2 Recommendations and insights

Based on the findings of this study, the following recommendations can be provided for policy-making:

(1)  Starting from policies and skill training, it is recommended to assist returning rural laborers in finding better employment and entrepreneurial opportunities.

Considering the local circumstances, it is recommended to actively develop certain industries that can drive local economic growth and provide more employment opportunities for returning laborers. Furthermore, it is suggested to establish a database of information on returning rural laborers, which can provide them with more employment information and relevant technical training. This will help enhance the human capital of returning laborers and enable them to secure better employment opportunities. It is also recommended to provide relevant information and policy support for rural laborers who choose to start businesses upon returning to their hometowns. Implementing the outcomes of these entrepreneurial endeavors and creating a demonstration effect can attract more talented and capable rural laborers to engage in entrepreneurship. This approach will not only generate more employment opportunities but also foster better entrepreneurial prospects for returning rural laborers. After discussing endogeneity, the results of this article remain robust.

(2)  Promoting and improving the new rural social pension insurance system should be encouraged, along with gradually supplementing and refining the supporting social security system.

Efforts should be intensified to promote the new rural social pension insurance system, gradually increase the level of pension benefits, and ensure a basic income for older adult individuals in rural areas. This will reduce their dependence on financial support from their children. Additionally, it is important to improve the rural medical security system, lowering healthcare expenses for older adult individuals in rural areas. By doing so, they will have the confidence and ability to seek medical treatment and maintain their overall health.

(3)  Efforts should be made to promote filial piety culture through public education. It is important to encourage children to utilize existing resources and actively fulfill their obligations to support their parents.

It is crucial to promote a positive and proactive culture of filial piety. This includes strengthening publicity and education efforts targeted at rural laborers, emphasizing the importance of maintaining strong connections with their parents regardless of whether they are working away or have returned home. Encouraging regular communication, expressing greetings and care, and fulfilling one’s obligations of support are vital. It is essential to avoid conflicts and rivalry with older adult parents, striving to enhance their quality of life for better happiness and improved health.

## Data availability statement

The raw data supporting the conclusions of this article will be made available by the authors, without undue reservation.

## Ethics statement

Written informed consent from the patients/ participants OR patients/participants legal guardian/next of kin was not required to participate in this study in accordance with the national legislation and the institutional requirements.

## Author contributions

HG: Conceptualization, Investigation, Methodology, Project administration, Software, Writing – original draft. SB: Funding acquisition, Investigation, Methodology, Project administration, Resources, Software, Supervision, Writing – review & editing. ZhW: Conceptualization, Investigation, Methodology, Project administration, Writing – original draft. ZiW: Formal analysis, Methodology, Project administration, Writing – review & editing.
